# CaroRite™ for improving skin health and the visible signs of aging - a randomised, double-blind, placebo-controlled trial

**DOI:** 10.3389/fragi.2026.1858090

**Published:** 2026-09-21

**Authors:** Amanda Rao, Janice Pellow, David Briskey

**Affiliations:** RDC Clinical, Brisbane, QLD, Australia

**Keywords:** CaroRite, carotenoids, dermal thickness, skin aging, skin health, TEWL (transepidermal water loss)

## Abstract

**Background:**

There is an increasing need to identify novel ingredients that can be safely utilized to enhance skin function and mitigate skin aging. Carotenoid supplementation shows promise in improving skin health and reducing signs of aging, owing to its antioxidant and anti-inflammatory properties.

**Aim:**

This randomised, double-blind, placebo-controlled study aimed to investigate the efficacy of a carotenoid supplement, CaroRite™, on skin health and the visible signs of aging in healthy females.

**Methods:**

Eighty female participants aged 40–55 years were recruited and randomised to receive either CaroRite™ (containing 28–30 mg mixed carotenoids) or a placebo. Participants were instructed to take one capsule daily for 12 weeks. Outcome measures included visible signs of aging (wrinkles, skin elasticity, hydration, sebum, pigmentation, pore size, and temperature), transepidermal water loss (TEWL), dermal thickness, skin carotenoid levels, dietary carotenoid intake, and safety.

**Results:**

No significant between-group differences were observed for visible signs of aging parameters (p > 0.05). At week 12, significant between-group differences from baseline were observed for TEWL (CaroRite™ −4.58 ± 9.43 vs. placebo 0.34 ± 6.73; p = 0.011) and dermal thickness (CaroRite™ 139.50 ± 220.21 μm vs. placebo 43.90 ± 225.97 μm; p = 0.036). Skin carotenoid levels increased by 48.1% in the active group (CaroRite™ +167.09 ± 117.23 vs. placebo 9.42 ± 47.16; p < 0.001), despite no significant differences in dietary carotenoid intake between groups.

**Conclusion:**

Daily oral supplementation with CaroRite™ did not reduce the visible signs of ageing after 12 weeks, however notable improvements in skin carotenoid status, TEWL and dermal thickness was found. CaroRite™ demonstrated good bioavailability and was well tolerated. Overall, these findings support the use of CaroRite™ as part of a comprehensive approach to potentially improving skin health, particularly for enhancing skin carotenoid status and supporting underlying structural and barrier function.

**Clinical Trial Registration:**

ClinicalTrials.gov, Identifier NCT06878001.

## 1 Introduction

Skin aging is a natural and biological phenomenon that occurs because of many complex factors. There are both intrinsic and extrinsic mechanisms that lead to the structural and physiological changes associated with aging. Intrinsic aging is also known as chronological aging, and is largely dependent on genetics, metabolic processes and hormonal shifts. Extrinsic aging, which is often referred to as photoaging, is the result of environmental factors such as ultraviolet (UV) radiation, smoking, and pollution. The two types of skin aging have several overlapping molecular mechanisms including the formation of reactive oxygen species (ROS), which damages cellular components such as proteins, lipids, and DNA, and results in cellular dysfunction and senescence. Intrinsic aging disrupts skin integrity and results in dry, thinning skin, fine wrinkles, decreased elasticity and skin sagging. Extrinsic aging is linked to the development of coarse wrinkles, loss of elasticity, and alterations in pigmentation (Shin et al., 2023).

The skin is the most visible organ, and as such, displays the most observable signs of human aging (Liang et al., 2023). The skin’s appearance has a profound influence on self-esteem, social interactions and quality of life, and consequently many people desire to have healthy and youthful-looking skin (Clatici et al., 2017). The signs of skin aging tend to be most notable on the face, neck, forearms and lower limbs, and these are also the areas where age-related skin diseases commonly occur. The various aging processes result in degeneration of the skin’s structural stability and functional integrity. Thinning of the epidermis has a significant impact on the skin’s ability to function as an effective protective barrier, while thinning of the dermal layer, which is primarily due to the loss of collagen, contributes to various skin conditions such as fragile skin, poor wound healing, skin infections and an increased risk for developing cancer. Aging skin is therefore more than an aesthetic concern, as it contributes to various dermatological issues (Liang et al., 2023; Stanescu et al., 2025; Quan, 2023).

In addition to the gradual loss of collagen and elastic fibres that occurs with advancing age, hormonal shifts during perimenopause and menopause can cause significant changes in skin appearance within a relatively short period of time. Oestrogen stimulates the production of collagen by fibroblasts in the extracellular matrix (ECM), as well as prevents age‐related dryness and transepidermal water loss (TEWL) by promoting the production of hyaluronic acid. As oestrogen levels decline, collagen production is impaired and its degradation accelerated, negatively affecting the structural integrity of the ECM (Viscomi et al., 2025). It is estimated that skin collagen levels in females decline around 30% during the first 5years of menopause and a further 2% every year thereafter (Fabi et al., 2026). These hormonal changes collectively contribute to skin thinning, dryness, decreased elasticity, wrinkles, and reduced vascularity, culminating in impaired skin barrier function, poor wound healing and more prominent signs of aging (Viscomi et al., 2025).

Owing to this, there is a growing interest in naturally derived geroprotectors, which are high performing interventions that slow, prevent or reverse aging-related decline and extend the lifespan by targeting the key mechanisms of aging (Klinngam et al., 2025). There is a need to investigate novel ingredients that can be safely implemented to improve skin function and reduce the visible signs of aging (Meléndez-Martínez et al., 2019).

Nutrition plays a critical role in skin health and appearance (Meléndez-Martínez et al., 2019), with carotenoids representing a key group of bioactive compounds. These tetraterpenoid pigments—responsible for red, yellow, and orange coloration—are found naturally in many fruits and vegetables, as well as in certain herbs, legumes, grains, and egg yolk, and are also consumed as dietary additives (colourants) and supplements (Meléndez-Martínez et al., 2019; Shanaida et al., 2025). Carotenoids have been widely studied for their antioxidant and anti-inflammatory properties. They show promise as a cosmeceutical addressing skin aging and skin health, as well as a nutraceutical that prevents disease and promotes health (Shanaida et al., 2025). Many studies have shown that greater dietary intake of carotenoids is associated with a reduced risk for developing cancer, cardiovascular disease, diabetes, neurodegenerative diseases, and skin and eye disorders (Meléndez-Martínez et al., 2019; Madore et al., 2023). Antioxidants play an important role in preventing oxidative damage to structural components in the skin improving skin health. Carotenoids produce their antioxidant activity by directly neutralizing free radicals, as well as via activating the body’s own antioxidant defense system, by promoting the expression of superoxide-dismutase, catalase, and glutathione-peroxidase (Stanescu et al., 2025).

Ingested carotenoids accumulate in the skin (Stanescu et al., 2025), where they contribute to photoprotection and skin structure. Higher dietary intake and tissue levels of carotenoids have been associated with reduced premature skin aging, likely due to their ability to protect against UV radiation and support collagen production (Shanaida et al., 2025; Singh et al., 2025). Carotenoids in the skin absorb specific wavelengths of UV light and dissipate the absorbed energy as harmless heat, thus preventing UV damage and subsequent photoaging and carcinogenesis (Stanescu et al., 2025). Various factors can affect the levels of carotenoids in the skin, such as gender, age, health status, UV and infrared radiation from sunlight, smoking, alcohol consumption and illness (Shanaida et al., 2025). Carotenoids exert their skin antiaging effects through a synergistic network of biological mechanisms providing antioxidative, anti-inflammatory, photoprotective, immunoregulatory and structural mechanisms that promote skin health and integrity and slow skin aging (Stanescu et al., 2025). Carotenoids also promote healing in skin conditions such as acne, psoriasis, and eczema (Meléndez-Martínez et al., 2019).

Sufficient dietary intake of carotenoids may be difficult for some, owing to limited availability of good quality food sources and high cost; therefore, supplements are available as a suitable alternative to consuming high volumes of carotenoid rich foods. CaroRite™ is a dietary supplement containing a blend of five carotenoids: lutein, zeaxanthin, α-carotene, β-carotene, and lycopene. The effects of CaroRite™ have previously been evaluated in two clinical trials. The first study evaluated the effects of CaroRite™ on macular pigment optical density (MPOD) and central foveal thickness in 93 healthy adults for 180 days. CaroRite™ produced a statistically significant increase in MPOD compared with placebo (Mehkri et al., 2024). The second study explored the effects of CaroRite™ supplementation on levels of oxidative stress marker eight-iso-PGF2α (a type of F2-Isoprostane), mental and physical wellbeing and quality of life in 77 adults aged 18–60 years. CaroRite™ supplementation of 28 mg daily improved perceived psychological wellbeing in older participants while greater reductions in oxidative stress markers were observed in younger individuals (Srivastava et al., 2026). There have been no studies conducted to date evaluating the efficacy of CaroRite™ on skin health and aging. Therefore, this study aimed to investigate the efficacy of CaroRite™ administered orally for 12 weeks on skin health and the visible signs of aging in healthy females aged 40–55 years old.

## 2 Materials and methods

### 2.1 Study design

This was a 12-week, randomised, double-blind, placebo-controlled, parallel group clinical study, conducted in Brisbane, Australia, between July and December 2025. Those who passed screening gave their written consent prior to participating in the study. Ethical approval was obtained from Bellberry Limited, Human Research Ethics Committee on the 16th of December 2024, approval number 2024–09-1202. This study was prospectively registered with ClinicalTrials.gov (NCT06878001). The age range of 40–55 years was set to include women with moderate signs of photoaging and is based on the age ranges of similar previously published studies (Rao et al., 2025; Cho et al., 2010; Tarshish et al., 2023).

### 2.2 Randomisation

Randomisation was performed by an independent person not involved in the conduct of the trial using Random Allocation Software (Sealedenvelope.com.au), ensuring that the investigators and participants were blinded to the product allocation. Enrolled participants were randomly allocated in a 1:1 ratio to one of two groups, receiving either the active or placebo product. The statistician was blinded during the data analysis.

### 2.3 Study intervention

The active softgel capsules (CaroRite™) contained 28–30 mg mixed carotenoids and 170–172 mg sunflower oil. The mixed carotenoids in the active formulation consisted of β-carotene (cis and trans) (24.0%–25.0%), α-carotene (0.5%–1.0%), lycopene (21.0%–22.0%), lutein (44.0%–45.0%), and zeaxanthin (8.5%–9.0%). The placebo arm received identical capsules containing 200 mg sunflower oil only. Both the active and placebo products were supplied by Bio-gen Extracts (India). The dosage and duration of supplementation chosen for this study were based on the findings of previous clinical trials involving carotenoid supplementation. One study showed that a dosage of 30 mg/day of *β*-carotene taken for 90 days (12 weeks) demonstrated anti-photo-aging effects in healthy females (Cho et al., 2010), while in another study, 12-week supplementation with zeaxanthin improved facial lines, wrinkles and hydration (Schwartz et al., 2016). In addition, CaroRite™ was found to produce positive antioxidant effects at a dosage of 30 mg in a previous study (Srivastava et al., 2026).

Both products (active and placebo) were manufactured to GMP standards and packed in the same type of container and labelled identically (except for the product number) in an Australian Therapeutic Goods Administration (TGA) licenced facility. Each softgel consisted of a dark outer shell to mask the contents and maintain the blind nature of the study. Participants were instructed to take one capsule daily with or immediately after a meal for 12 weeks.

### 2.4 Eligibility criteria

Eighty participants from Brisbane, Queensland who met the screening criteria were enrolled into the trial. Inclusion criteria were generally healthy females aged 40–55 years with a body mass index (BMI) of 18.5–29.9 kg/m^2^. Exclusion criteria were having a serious or unstable illness; current malignancy (excluding Basal Cell Carcinoma) or treatment for malignancy in the previous 2 years; regular intake of carotenoid containing medication or supplements (beta-carotene, lycopene, lutein, zeaxanthin, astaxanthin) in the previous 2 months; cosmetic surgery or procedures including Botox and other injectables, microdermabrasion and laser treatments on the face in the previous 3 months; use of medications for acne or other skin conditions including topical retinoids (e.g., Retin-A, Retrieve), oral retinoids such as isotretinoin, benzoyl peroxide, or use of alpha hydroxy acids, serum and chemical peels within the past month; active smokers, nicotine use or drug (prescription or illegal substances) abuse; chronic past and/or current alcohol use (>14 alcoholic drinks week); pregnant or lactating women; allergic to any of the ingredients in the study products; and current participation in another clinical trial or in a clinical trial that may affect skin health during the past 1 month. Participants agreed not to change their regular skincare routine and abstain from using cosmetic injectables during the study.

### 2.5 Outcome measures

Outcome measures were completed at baseline, week 6 and week 12. The primary outcome of this study was the change from baseline to the end of the study period in the visible signs of aging including wrinkles, skin elasticity, hydration, sebum, pigmentation, pore size and temperature. These were assessed using the A-One Smart Pro (Bomtech Electronics Co. Ltd., Seoul, Korea). Prior to the skin assessment, participants removed all make-up using make-up removal wipes, hair was cleared away from the face, and time allowed for acclimation to the ambient temperature of the clinic. The clinic was climate controlled at a temperature of 23 °C, and had standardised lighting conditions. The A-One Smart Pro uses a high-resolution camera to take photographs of the skin under normal, UV and polarized lighting. The system uses integrated software to align facial landmarks and applies algorithms to analyse various skin parameters on these captured images, including skin surface texture, moisture distribution, elasticity, vascular patterns, pigmentation and sebum levels. For elasticity, the probe measured the skin’s firmness and ability to immediately bounce back when compressed. The system’s AI software cross-referenced the visual image with this value to determine a numerical elasticity score. Wrinkles were measured as thin or thick and rated on a scale of 1–10 in each of the following anatomical areas: forehead, eyes, mouth and nose. These measurements were then compared against an internal age-matched reference database to generate a wrinkle score that reflected both absolute and relative severity. An attached probe was used to measure skin temperature and moisture. The A-One Smart Pro has been used in a previous clinical study assessing skin aging (Rao et al., 2025; Ha et al., 2017; Lee et al., 2015).

Secondary outcomes included changes in transepidermal water loss (TEWL), dermal thickness, skin carotenoid levels, and carotenoid intake. Safety monitoring consisted of assessing adverse events at each study visit. TEWL and dermal thickness were analysed using the DermaLab Combo® system (Cortex Technology, Hadsund, Denmark). The DermaLab Combo® comprises of a central unit that supports multiple assessment probes. The TEWL probe, an open‐chamber device, measures vapour pressure gradient at the skin surface. Dermal thickness was determined using a pulsed 20 MHz B‐mode high frequency ultrasound. The TEWL and ultrasound probes were gently placed perpendicular to the skin surface without any pressure. Previous studies have reported that the DermaLab Combo® is a reliable tool for measuring both TEWL and skin thickness (Rayner et al., 2017). Skin carotenoid levels were analysed using the Veggie Meter® analyser (Longevity Link Corporation, Utah, United States). The Veggie Meter® is a non-invasive device that uses pressure-mediated reflection spectroscopy (RS) for detecting and quantifying skin carotenoids in the skin. It connects to an interfaced laptop computer to display an individual’s “skin carotenoid score” on a histogram, with the x-axis illustrating the range of scores from 0 to 800, and the y-axis representing the reference population frequency (Radtke et al., 2021). The Veggie Meter® has been used in previous clinical trials evaluating carotenoid levels in various population groups (Radtke et al., 2021; May et al., 2020; Fultz et al., 2022; Caparello et al., 2023; Jilcott et al., 2023). The score breakdown for Veggie Meter® is: 0–299 (low carotenoid intake), 300–449 (moderate carotenoid intake), 450–800+ (high carotenoid intake). Generally, a score between 280 and 480 is considered healthy, though higher scores are considered more ideal. Each ∼100-unit increase roughly corresponds to an additional daily serving (1 cup) of fruits and vegetables (Di Noia and Gellermann, 2021). The self-administered Carotenoid Intake Questionnaire was used to monitor changes in dietary carotenoid intake during the study. The safety of CaroRite™ compared to placebo was evaluated by assessing adverse event (AE) reporting throughout the study period.

Once the participants completed all baseline measurements, they were instructed to take the allocated study capsules daily for 12 weeks. Participants returned to the clinic at week 6 and week 12 to repeat baseline measurements. Investigational product compliance was monitored throughout the study and participants returned any unused trial products at their final visit.

### 2.6 Sample size calculation and data analysis

Data was analysed using SPSS Statistics V30. The sample size calculation was based on a previous study of similar design (Mehkri et al., 2024). With 80% power and a significance level of 0.05, a sample size of 33 participants per group was required to detect a 10% difference between groups in wrinkle scores (4.0 ± 0.8 vs. 4.5 ± 0.8). To account for potential dropouts, 40 participants per group were enrolled. Data was analysed using a modified intent-to-treat (mITT) principle that included all randomised participants who had at least one post-baseline measurement for the primary outcome. Differences between groups were assessed using the t-test, with statistical significance set at p < 0.05. Additional analyses included a repeated-measures ANOVA. For between group differences, data was analysed using both absolute and change from baseline data. Any participant that presented a result considered an outlier (±2 SD away from the mean) was excluded from analysis. Baseline measurements identified as implausible following review of the participant’s longitudinal data and measurement consistency were excluded from analysis. For safety, the incidence of AEs was compared between each group.

## 3 Results

Eighty eligible participants were randomised to the CaroRite™ (n = 40) or placebo (n = 40) groups. The data from two participants in the active group was excluded from the mITT analysis as they were identified as falling outside the specified age eligibility range (40–55 years) at their baseline visit. Both participants had passed initial remote or telephone screening; however, verification of age was conducted at the baseline clinic appointment, at which point the deviation was identified, and they did not receive investigational product. Their baseline data was collected but not included in the analysis. See Figure 1 for the representation of the screening, allocation and analysis of study participants.

**FIGURE 1:**
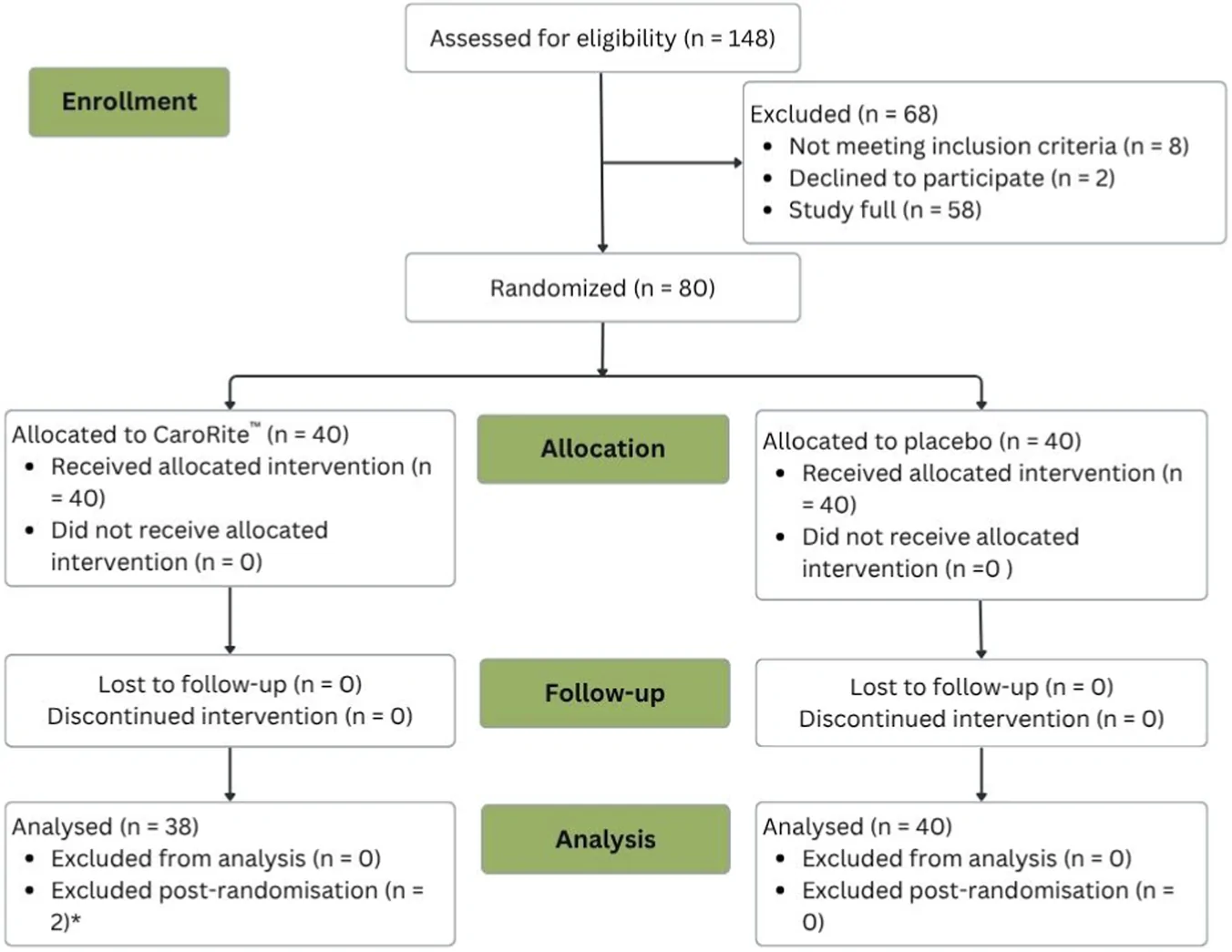
CONSORT Flow Chart. *Did not meet age eligibility criterion upon verification at baseline visit; no investigational product received; baseline data collected but not included in any analysis.

### 3.1 Demographic and baseline characteristics

There were no significant differences in demographic and baseline characteristics between groups (Table 1). Treatment adherence was high, with all participants taking at least 80% of prescribed doses. The mean compliance was 98.5% ± 0.10 in the CaroRite™ group and 102.0% ± 0.09 in the placebo group.

**TABLE 1:** Participant demographics and baseline characteristics.

Parameter	CaroRite™ (n = 40)	Placebo (n = 40)	p-value
Age (year) (SD)	47.7 (5.2)	48.0 (5.1)	0.779
Height (meter) (SD)	1.60 (0.1)	1.70 (0.1)	0.203
Weight (kilogram) (SD)	63.2 (9.5)	65.5 (9.5)	0.276
BMI (SD)	23.6 (2.9)	24.0 (3.2)	0.587

Values presented are mean (SD). SD, standard deviation.

### 3.2 Primary outcome–Visible signs of aging

Both groups had similar baseline scores for the various visible signs of aging, indicating that they were well matched. At both week 6 and week 12, there were no significant between group differences for any of the aging parameters measured (p > 0.05) (Table 2). There was a significant difference between groups in the change in elasticity scores in favour of the placebo group (CaroRite™ 5.97 ± 24.23, placebo 19.85 ± 34.80; p = 0.047; Cohen’s d = −0.46).

**TABLE 2:** Visible signs of aging.

Outcome	CaroRite™ (n = 38)			Placebo (n = 40)				CaroRite™ (n = 38)	Placebo (n = 40)	​
	Baseline	Week 6	Week 12	Baseline	Week 6	Week 12	*p-value	Δ	Δ	**p-value
Age	47.18 (4.64)	47.29 (4.59)	47.32 (4.57)	47.90 (4.97)	48.00 (4.97)	48.20 (4.98)	0.416	0.13 (0.34)	0.30 (0.46)	0.071
Skin age	48.66 (4.81)	48.76 (4.81)	48.79 (4.66)	49.58 (5.20)	49.55 (4.96)	49.73 (4.91)	0.391	0.13 (1.14)	0.15 (1.10)	0.942
Moisture level	34.92 (2.82)	36.08 (5.57)	40.08 (5.71)	34.35 (3.94)	35.20 (6.02)	38.90 (5.79)	0.368	5.16 (4.93)	4.55 (5.78)	0.618
Elasticity	52.69 (20.18)	47.03 (22.16)	52.89 (18.45)	45.04 (21.39)	47.75 (20.77)	51.38 (19.90)	0.727	5.97 (24.23)	19.85 (34.80)	0.047
Total wrinkles	4.08 (1.16)	4.31 (1.49)	4.13 (1.21)	3.95 (0.89)	4.09 (0.96)	4.17 (1.04)	0.878	0.05 (1.03)	0.21 (1.12)	0.505
Forehead lines	3.03 (1.26)	3.21 (1.56)	2.91 (1.00)	2.84 (0.87)	2.86 (0.97)	2.83 (0.99)	0.727	−0.12 (1.27)	0.00 (0.65)	0.608
Glabellar lines	3.49 (1.15)	3.82 (1.28)	3.80 (1.32)	3.59 (1.05)	3.71 (1.00)	3.80 (0.84)	0.979	0.30 (0.89)	0.21 (0.83)	0.622
Crow’s Feet	3.17 (0.97)	3.36 (1.04)	3.31 (0.84)	3.23 (0.74)	3.25 (0.88)	3.51 (1.15)	0.386	0.15 (0.62)	0.28 (1.10)	0.515
Nasolabial folds	2.52 (0.82)	2.72 (0.89)	2.82 (0.99)	2.49 (0.86)	2.73 (1.04)	2.71 (0.74)	0.563	0.31 (0.77)	0.22 (0.51)	0.548
Sebum U-zone	551.05 (427.11)	629.03 (610.11)	580.32 (383.49)	467.53 (284.48)	539.73 (335.52)	501.15 (336.36)	0.340	14.00 (347.19)	33.62 (221.71)	0.768
Sebum T-zone	528.39 (715.05)	446.03 (529.06)	382.42 (466.15)	466.75 (509.80)	429.93 (608.35)	436.93 (588.40)	0.651	−145.97 (669.95)	−29.83 (405.25)	0.361
Pigmentation	3.94 (1.25)	4.07 (1.25)	4.05 (1.19)	4.31 (0.73)	4.15 (0.85)	4.23 (1.00)	0.475	0.10 (0.66)	−0.08 (0.55)	0.190
Temperature	20.68 (1.69)	20.89 (1.16)	20.82 (0.95)	19.80 (1.52)	20.70 (1.20)	20.40 (1.43)	0.134	0.13 (1.92)	0.60 (1.89)	0.281
Pore size	6.38 (0.78)	6.61 (0.48)	6.62 (0.47)	6.55 (0.38)	6.57 (0.42)	6.61 (0.32)	0.902	0.24 (0.63)	0.06 (0.27)	0.111

Values presented are mean (SD); Δ = change over time; *difference between groups at week 12; **difference between groups in change from baseline and week 12.

### 3.3 Secondary outcomes

#### 3.3.1 TEWL

Both groups showed a decrease in TEWL scores at week 6 and 12, which was numerically higher in the CaroRite™ group (Table 3), however there were no significant differences between groups at any time point. Notably, there was a statistically significant improvement in the change in TEWL score in the active group compared to placebo (CaroRite™ −4.58 ± 9.43, placebo 0.34 ± 6.73 [mean difference −4.24]; p = 0.011; Cohen’s d = −0.60, *post hoc* power ∼75%) (Table 3; Figure 2). This change corresponded to a 31.3% decrease in TEWL from baseline in the CaroRite™ group. A repeated-measures ANOVA was performed incorporating Week 6 and Week 12 assessments. A significant overall treatment effect was observed (F (1,73) = 5.559, p = 0.021), while neither the effect of time (p = 0.177) nor the treatment × time interaction (p = 0.316) was significant.

**TABLE 3:** TEWL, dermal thickness and skin Carotenoid scores.

Outcome	CaroRite™ (n = 38)				Placebo (n = 40)					
	Baseline	Week 6	Week 12	*Δ*	Baseline	Week 6	Week 12	*Δ*	*p-value	**p-value
TEWL	14.65 (9.14)	12.37 (6.22)	10.07 (4.44)	−4.58 (9.43)	12.58 (9.17)	11.98 (7.97)	11.34 (6.24)	0.34 (6.73)	0.301	0.011
Dermal thickness	1170.03 (281.24)	1323.66 (252.85)	1323.29 (263.33)	139.50 (220.21)	1212.40 (288.52)	1249.21 (229.16)	1269.03 (237.46)	43.90 (225.97)	0.343	0.036
Skin Carotenoid Scores	347.32 (106.95)	465.99 (120.90)	514.40 (129.85)	167.09 (117.23)	353.75 (99.53)	371.22 (100.07)	363.12 (99.40)	9.42 (47.16)	<0.001	<0.001

Values presented are mean (SD); Δ = change over time; *difference between groups at week 12; **difference between groups in change from baseline and week 12.

**FIGURE 2:**
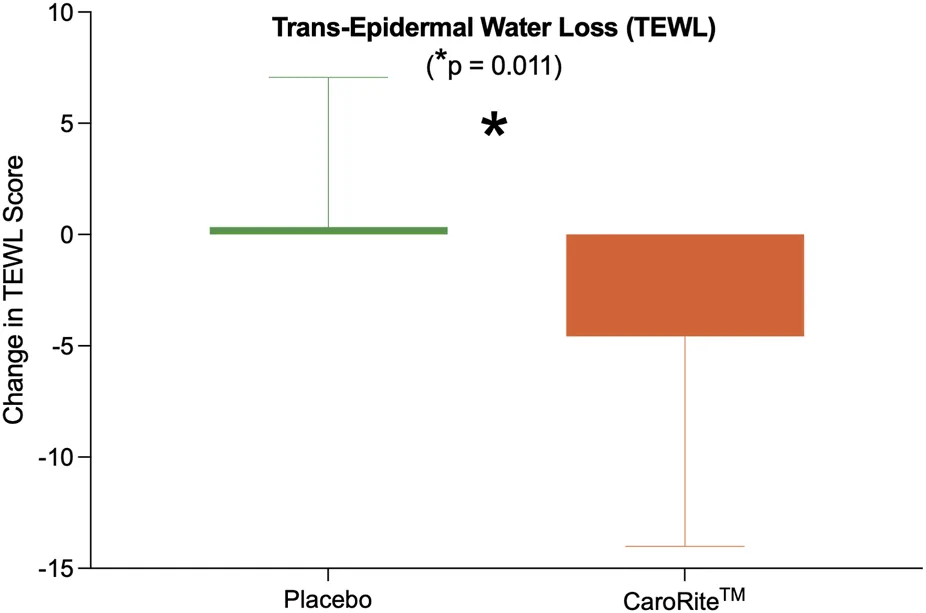
Change in TEWL between baseline and week 12.

#### 3.3.2 Dermal thickness

At week 12, the CaroRite™ group showed a greater mean increase in dermal thickness scores compared to placebo at both week 6 and week 12 (Table 3) with no statistically significant difference between groups. The difference in the change in score from baseline between groups was statistically significant (CaroRite™ 139.50 ± 220.21 μm, placebo 43.90 ± 225.97 μm; mean difference 95.6 μm, p = 0.036; Cohen’s d = 0.43, post hoc power ∼46%) (Table 3; Figure 3). This change corresponded to an 11.9% increase in dermal thickness from baseline in the CaroRite™ group compared to a 3.6% increase in the placebo group. A repeated-measures ANOVA showed a significant overall treatment effect (F (1,71) = 5.071, p = 0.027), while neither the effect of time (p = 0.483) nor the treatment × time interaction (p = 0.383) was significant.

**FIGURE 3:**
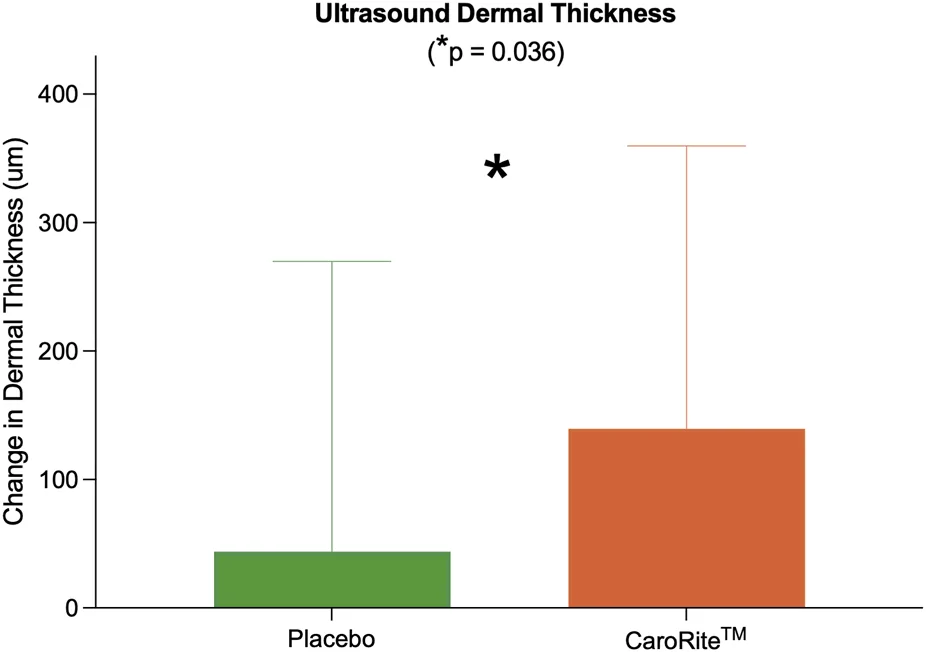
Change in dermal thickness between baseline and week 12.

#### 3.3.3 Skin carotenoid scores

Baseline skin carotenoid levels were similar in the groups (p = 0.785). The CaroRite™ group had a highly significant increase in skin carotenoid scores compared to placebo at week 6 (CaroRite™ 118.68 ± 88.58; placebo 17.93 ± 50.97; mean difference 100.75, p < 0.001) and week 12 (CaroRite™ 167.09 ± 117.23; placebo 9.42 ± 47.16; mean difference 157.67, p < 0.001; Cohen’s d = 1.78, post hoc power of >99%). This change in score from baseline equates to a 48.1% increase in carotenoid levels in the CaroRite™ group compared to a 2.6% increase in the placebo group (Figure 4). For carotenoid concentration, repeated-measures ANOVA showed significant effects of treatment (p < 0.001), time (p = 0.004), and treatment × time interaction (F (1,75) = 56.74, p < 0.001) were observed, indicating a differential response over time between treatment groups.

**FIGURE 4:**
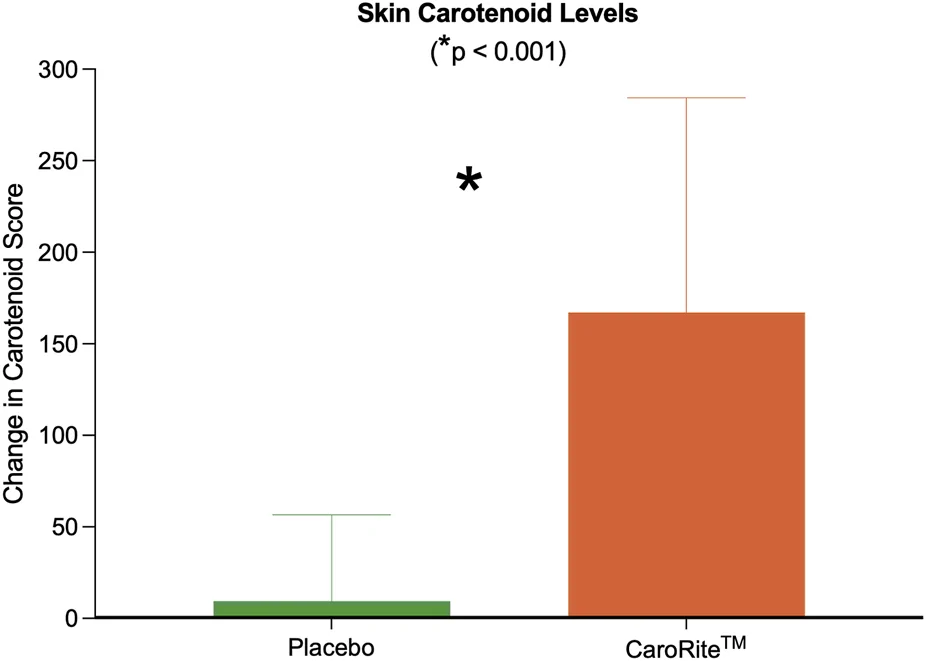
Change in skin Carotenoid levels between baseline and week 12.

#### 3.3.4 Carotenoid Intake Questionnaire

At baseline both groups reported a similar intake in carotenoids from their diet (Table 4). Both the CaroRite™ and placebo groups had an increase in the quantity of carotenoids consumed in the diet at week 12 (CaroRite™ +4.08, placebo +4.34), however there was no significant difference found between the groups (p = 0.222).

**TABLE 4:** Carotenoid intake Questionnaire scores.

Timepoint	CaroRite™ (n = 38)	Placebo (n = 40)	*p-value
Baseline	30.60 (14.45)	35.25 (15.25)	0.176
Week 12	34.68 (18.11)	39.59 (16.78)	0.222

* difference between groups at week 12.

#### 3.3.5 Safety

Thirteen adverse events (AEs) were reported among 13 participants (CaroRite™ n = 5; placebo n = 8) and were classified as mild (n = 12; 92.3%) or moderate (n = 1; 7.6%) in severity. Mild AEs are defined as transient, do not interfere with usual activities of daily living, and may require only minimal intervention. Moderate AEs interfere with usual activities of daily living, cause discomfort but poses no significant or permanent risk of harm and are usually alleviated with additional specific therapeutic intervention. Four (30.7%) adverse events were classified as ‘not related’ to the treatment, and a further four (30.7%) as unlikely related. The most frequently reported adverse events were self-limiting and gastrointestinal in nature, e.g., bloating, reflux, eructation, loose stools (CaroRite™ n = 3; placebo = 2). There were no serious adverse events reported during the study and no participant withdrawals due to adverse events suggesting good tolerability of the supplement.

## 4 Discussion

Carotenoids are natural, safe and versatile compounds that have been shown to play a key role in promoting health and supporting healthy aging, primarily through their antioxidant and anti-inflammatory effects (Stanescu et al., 2025; Meléndez-Martínez et al., 2019). Their value in the context of nutricosmetics continues to grow as studies show that they have the potential to prevent premature skin aging, support repair processes and enhance skin health (Stanescu et al., 2025; Meléndez-Martínez et al., 2019; Ma et al., 2025). This study aimed to assess the efficacy of oral CaroRite™ supplementation compared to a placebo, on skin health and the visible signs of aging in females aged 40–55 years over 12 weeks. The results showed that while there were no significant differences found in the visible signs of aging (including wrinkles, hydration, sebum, pigmentation, pore size, and temperature), CaroRite™ significantly reduced TEWL by 31.3% and increased dermal thickness by 11.9% over the 12-week study period. In addition, skin carotenoid levels increased significantly in the CaroRite™ group from week 6 onwards, with an increase in score from baseline to week 12 of around 48.1%, suggesting notable bioavailability of the supplement. Overall, CaroRite™ was well tolerated with the incidence of adverse events being similar between groups. The most frequently reported adverse events were mild, self-limiting and gastrointestinal in nature.

These results are comparable to other clinical trials involving carotenoid supplementation. A study by Palombo et al. (2007) found that 12 weeks of supplementation with lutein and zeaxanthin in oral, topical or combined form increased elasticity and skin hydration as well as provided significant antioxidant protection in women aged 25–50 years (Palombo et al., 2007). Schwartz et al. (2016) showed that an oral zeaxanthin-based dietary supplement effectively reduced wrinkle count and wrinkle severity compared to placebo, and when used in combination with a topical serum, significant improvements in skin hydration and other skin health parameters were found (Schwartz et al., 2016). Darvin et al. (2011) evaluated the effects of topical, systemic and combined antioxidant treatments on skin carotenoid levels in healthy female participants aged 21–72 years. The results showed that topically applied carotenoids were not as effective at increasing skin carotenoid levels as oral carotenoids, as they were stored in the stratum corneum for only a short period of time while the orally administrated carotenoids were stored in body fat tissues and then slowly released into the skin layers over time. The combination of topical and oral carotenoids produced the highest accumulation (Darvin et al., 2011). In a 90-day clinical study by Cho et al. (2010), β-carotene (30 mg/day) significantly increased procollagen type I levels by 4.4-fold compared to baseline, and improved wrinkles and elasticity in photo-aged skin in healthy females over the age of 50 (Cho et al., 2010). Meinke et al. (2012) demonstrated that a formulation containing lutein, β-carotene, zeaxanthin, lycopene, α-carotene and cryptoxanthin taken over 8 weeks resulted in a significant increase in cutaneous carotenoids, and increased skin lipids in healthy participants compared to the placebo group, but only significantly for ceramide (Meinke et al., 2013). A systematic review and meta-analysis by Zhang et al. (2024) which included 21 clinical trials reported that tomato and lycopene supplementation protected against photoaging, increased skin thickness and density, and improved pigmentation and the overall appearance of the skin (Zhang et al., 2024).

Achieving optimal skin hydration is key to improving skin health and appearance, maintain elasticity and function. With increased age, the ability of the skin to retain water is impaired as a result of decreased lipid synthesis, and impaired barrier integrity (Stanescu et al., 2025). TEWL is a widely used objective measurement for assessing skin barrier function. Elevated TEWL is indicative of skin barrier dysfunction, and importantly, is a feature of skin diseases such as atopic dermatitis, contact dermatitis, psoriasis and ichthyosis (Alexander et al., 2018). The improvement in barrier function (reduced TEWL) seen with CaroRite™ consumption may result from the carotenoids antioxidant protection of lipid membranes in the stratum corneum (Stanescu et al., 2025). Studies have shown that certain carotenoids (such as β-carotene, lutein, and astaxanthin) improve skin hydration via several mechanisms, namely, by strengthening the stratum corneum barrier (by regulating skin ceramide and filaggrin protein levels), regulating the function of cellular water channels known as aquaporins, preserving ECM, and regulating enzymes that degrade structural proteins like collagen. Carotenoids also maintain skin hydration by enhancing the production of hyaluronic acid in the ECM of the dermis, which plays a key role in tissue hydration, water retention and turgor (Stanescu et al., 2025; Tarshish et al., 2023). Improved barrier function is associated with better skin hydration retention, reduced sensitivity, and protection against environmental irritants and allergens (Baker et al., 2023). While established minimum clinically important difference (MCID) thresholds for TEWL exist primarily in the context of inflammatory skin conditions such as atopic dermatitis, where absolute changes of this magnitude are considered clinically relevant, currently there is no validated MCID for TEWL in healthy, non-diseased aging populations. Whether a reduction of 31.3% translates to a perceptible improvement in skin comfort or dryness in a healthy aging population cannot be confirmed from the current data, as no patient-reported outcome measure of skin changes was performed in this study.

Collagen is vital for skin health as it provides tensile strength and elasticity to the dermis (Shin et al., 2019). Carotenoids are known to protect against oxidative damage from ROS, which degrade collagen, elastin, and other structural proteins in the dermis. By neutralizing ROS, carotenoids may preserve existing collagen and support new collagen synthesis, leading to increased skin thickness. β-Carotene, lutein, astaxanthin, and lycopene in particular have been shown to support the production of collagen and inhibit its breakdown, thereby preserving the skin’s structural integrity (Stanescu et al., 2025). In this study, CaroRite™ supplementation significantly increased dermal thickness on ultrasound, which is supportive of enhanced collagen deposition in the ECM. Dermal thickness as measured by high-frequency ultrasound, is a structural biophysical parameter, not a clinical outcome. An 11.9% increase in dermal thickness over 12 weeks is directionally consistent with known carotenoid mechanisms and represents a biologically meaningful signal, however, it did not equate to a perceptible improvement in skin appearance and the visible signs of ageing. The structural change detected on ultrasound had not yet translated to measurable surface-level effects within the 12-week study period.

The benefits resulting from carotenoid intake depends not only on the amount consumed but also their bioavailability after ingestion. Carotenoids encounter several challenges in terms of their bioavailability owing to their lipophilic nature. In the digestive tract, carotenoids need to be emulsified and incorporated into micelles for intestinal absorption, however, this process can be subject to degradation, generally resulting in low bioavailability (Jilcott et al., 2023). Typically, only around 5%–30% of the ingested quantity of carotenoids are absorbed. Factors such as the presence of dietary fats, the type of food matrix, genetic factors, nutrient status of the individual, and individual digestive variations influence the absorption of carotenoids (Ma et al., 2025; Molteni et al., 2022). Daily CaroRite™ supplementation was shown to rapidly and significantly increase skin carotenoid levels as early as 6 weeks, which suggests efficient systemic absorption and delivery of carotenoids to skin tissue confirming their bioavailability and validating the formulation design and delivery system. Such rapid enrichment of skin antioxidant status may represent an important mechanistic step preceding improvements in barrier function and dermal structure.

Despite the growing evidence encouraging the integration of carotenoids into personalized anti-aging protocols for skincare, there are ongoing debates regarding their optimal dosage (Stanescu et al., 2025). Carotenoids are not considered essential nutrients, and as such there are no specific deficiency symptoms associated with low carotenoid intake (Eroglu et al., 2024) and no standardised recommended dietary intake guidelines (Böhm et al., 2021). In general, carotenoid supplementation is widely regarded as safe, with no toxicity of high intakes of carotenoids reported (Eroglu et al., 2024). Under specific conditions and in high doses, carotenoids may exert unwanted effects. One concern with excessive intake is carotenoderma, a harmless condition caused by carotenemia and which results in skin yellowing (Ma et al., 2025), however this was not found in the current study. The results of the current study showed that CaroRite™ supplementation was safe and well tolerated, and these findings contribute to the body of knowledge regarding optimal dosage and delivery systems for carotenoids.

Although no significant changes were observed in overall skin appearance, the observed reduction in TEWL and increase in dermal thickness suggest that CaroRite™ supplementation may positively influence underlying skin health. Improvements in barrier function and collagen architecture are well-recognised contributors to skin resilience and long-term dermal stability, which may precede detectable changes in visible ageing markers. Collectively, these findings support the potential role of CaroRite™ supplementation as a nutritional strategy to promote skin health and potentially mitigate the progression of age-related skin changes with sustained use.

## 5 Limitations

There were several limitations in this study. While wrinkle scores showed minimal changes in both groups, there were no statistically significant differences. Wrinkle formation is a complex process involving not only dermal thinning but also chronic muscle contractions, gravitational effects, and accumulated photodamage. Structural changes, such as sagging and wrinkling, originate in alterations in the dermis, which is a tissue with slow turnover, and therefore take time to develop (Fanian et al., 2013). While previous studies conducted on carotenoids (β-carotene and zeaxanthin) over 12 weeks showed positive results (Schwartz et al., 2016; Cho et al., 2010), it is possible that the 12-week timeframe of this study may not have been sufficient time for structural changes in dermal thickness to be observable reductions in established wrinkles, particularly in a population of healthy females with moderate photoaging. Longer intervention periods may be necessary to observe measurable changes in wrinkle depth and appearance. Given the lack of observable differences between groups in the primary outcome, which contrasts with other carotenoid studies (Palombo et al., 2007; Schwartz et al., 2016; Darvin et al., 2011; Cho et al., 2010; Meinke et al., 2013; Zhang et al., 2024), the study may have been underpowered to detect clinically meaningful changes in visible ageing outcomes. The *a priori* sample size calculation assumed a between-group difference in wrinkle scores of 0.5 points (4.0 vs. 4.5) with a shared SD of 0.8, yielding an assumed Cohen’s d of 0.625. This assumption was drawn from a previous CaroRite™ study (Mehkri et al., 2024), which evaluated macular pigment optical density and central foveal thickness, outcomes that are physiologically distinct from wrinkle scores. This reference was not ideally matched to the primary outcome of the current study, and that effect size assumptions derived from a different outcome domain introduce uncertainty into the power calculation. Another potential limitation is that the placebo effect may have occurred. While subjective measures are vulnerable to the placebo effect, this study reports objective means to measure ageing signs, mitigating the likelihood of the placebo effect occurring. The maintenance of usual reported baseline skincare routines and other behavioural modification could not be fully controlled and may have influenced the results of the study; however, participants were encouraged to report any changes in this regard. Dry and cold weather conditions have a marked effect on hydration levels and barrier function of exposed skin, particularly the face (Fanian et al., 2013), while hot humid weather causes the production of perspiration, increasing hydration levels, sebum secretion, TEWL, and greasiness while reducing skin pH [40]. Despite efforts to maintain a consistent testing environment (humidity and temperature), seasonal weather changes may have influenced the findings. Wrinkling and sagging however have not been shown to be readily influenced by seasonal changes (Fanian et al., 2013). Investigators had extensive training on the assessment equipment, however inter-rater variability amongst investigators may have influenced the results. While significant effects were observed for TEWL and dermal thickness, these findings should be interpreted in the context of multiple end-point testing and warrant confirmation in future studies. No patient-reported outcome measure of skin comfort or barrier-related symptoms was included in this study. Future studies should incorporate validated patient-reported outcomes alongside objective barrier measurements. Laboratory safety markers were not assessed in this study, therefore future trials should include comprehensive panels to monitor safety. The results of this study are limited to healthy females aged 40–55 years, with established skincare routines, and cannot be generalised to males, older populations, individuals with more severe photoaging or in those suffering from dermatological conditions and presents an avenue for future studies.

## 6 Conclusion

Oral daily CaroRite™ supplementation for 12-week did not produce notable changes in skin appearance or in the observable signs of ageing in healthy females aged 40–55 years, however, it significantly increased skin carotenoid levels, improved TEWL, and increased dermal thickness. These findings suggest that CaroRite™ may provide beneficial changes in antioxidant status, skin barrier function and dermal structure - key parameters necessary for optimal skin health. CaroRite™ also demonstrated excellent bioavailability and was well tolerated. Together, these findings support the use of CaroRite™ supplementation as part of a comprehensive approach to potentially improving skin health, particularly for enhancing skin carotenoid status and supporting underlying structural and barrier function. Future studies should consider longer intervention periods, evaluating other doses of CaroRite™ either alone or in combination with other nutrients, and in populations with more advanced photoaging.

## Data Availability

The raw data supporting the conclusions of this article will be made available by the authors, without undue reservation.
